# A Randomized, Double‐Blind, Parallel‐Group, Phase 1 Clinical Trial Comparing the Pharmacokinetic, Safety, and Immunogenicity of the Biosimilar HS016 and the Originator Adalimumab in Chinese Healthy Male Subjects

**DOI:** 10.1002/cpdd.816

**Published:** 2020-05-28

**Authors:** Guoying Cao, Jicheng Yu, Jufang Wu, Jingjing Wang, Yu Xue, Xiaoli Yang, Jing Zhang

**Affiliations:** ^1^ Phase I Clinical Trial Center Huashan Hospital Fudan University Shanghai China

**Keywords:** adalimumab, bioequivalence, immunogenicity, pharmacokinetics, safety

## Abstract

A comparison of the immunogenicity, safety, and pharmacokinetic properties of HS016 and its originator, adalimumab, was conducted in Chinese healthy male subjects. This was a phase 1 single‐center, randomized, parallel‐group double‐blind clinical trial. Chinese healthy male subjects (1:1) allocated to HS016 and adalimumab groups were treated with single subcutaneous injections (40 mg/0.8 mL). The pharmacokinetic equivalence of HS016 and adalimumab was assessed by (1) the area under the plasma concentration‐time curve (AUC) from time 0 to the last detectable drug concentration (AUC_0‐t_), (2) the AUC from time 0 extrapolated to infinity (AUC_0‐∞_), and (3) the maximum plasma concentration (C_max_). Other pharmacokinetic parameters (time to C_max_, apparent clearance, and half‐life), safety, and immunogenicity were also evaluated. A total of 136 subjects were randomly divided into HS016 (n = 68) or adalimumab (n = 68) groups. The geometric means of AUC_0‐t_, AUC_0‐∞_, and C_max_ were similar for HS016 and adalimumab. The 90%CIs of AUC_0‐t_ (87.2% to 106.1%), AUC_0‐∞_ (87.4% to 108.4%), and C_max_ (98.6% to 113.6%) were all within the prespecified bioequivalence criteria (80% to 125%). The incidence of treatment‐emergent adverse events (TEAEs) was similar in both groups, with most TEAEs being mild; only 3 (4.4%) subjects in the HS016 group experienced moderate TEAEs. No significant differences in the time to C_max_, apparent clearance, half‐life, and immunogenicity were detected. The pharmacokinetic profile of HS016 was equivalent to that of the originator, adalimumab, with similar safety and immunogenicity profiles. HS016 may be considered for assessment in the treatment of patients with ankylosing spondylitis.

The multifunctional cytokine tumor necrosis factor‐α (TNF‐α) is mainly produced by macrophages, monocytes, and activated T cells. It is secreted in both soluble and transmembrane forms and is known to play a vital role in the pathogenesis of immune‐mediated inflammatory disease.^1^, ^2^ Inhibitors that target TNF‐α bind not only to the transmembrane and soluble forms of TNF‐α but also to its precursor, thus blocking its interaction with the tumor necrosis factor receptor.^3^, ^4^ Therefore, biological agents that target TNF‐α can be used to treat various TNF‐α–related diseases such as rheumatoid arthritis (RA), septic shock, ankylosing spondylitis (AS), psoriasis, and other autoimmune diseases and to suppress the immunological rejection mechanisms underlying organ transplantation.^5^

The originator, adalimumab, is a recombinant human immunoglobulin (Ig) G1 monoclonal antibody (mAB) developed by Abbott Laboratories (Lake Bluff, Illinois) and has been marketed in the United States from 2003 and in China from 2010. The indications for the clinical use of adalimumab in China are moderate to severe active RA, severe AS, and chronic plaque psoriasis.^6^ The efficacy and safety of adalimumab have been widely verified, and it is 1 of the most commonly prescribed drugs for the treatment of RA and AS on a worldwide basis.^7^

Although biologics (etanercept, adalimumab, tocilizumab, infliximab) have shown significant improvements in efficacy and safety, the real‐world use of biological agents in China is lower due to their high cost and medical insurance reimbursement issues.^8^ As the expiration date of the patented originator adalimumab nears, physicians expect a biosimilar to have comparable properties with regard to safety, immunogenicity, and efficacy. Ideally, the biosimilar should be marketed at a lower price.^9^ The utilization rate of the biosimilar drug Yisaipu (Sunshine Guojian Pharmaceutical [Shanghai] Co, Ltd, Shanghai, China) for the treatment of RA in Chinese patients has been much higher than that of the original drug etanercept (Amgen, Thousand Oaks, California) (58.1% versus 6.1%),^10^ indicating the insistent demand for biosimilar drugs to treat tens of thousands of Chinese patients with AS and other immune diseases.

HS016, as a biosimilar drug equivalent to adalimumab, is a recombinant human mAB administered by injection that targets TNF‐α. It is virtually identical to adalimumab in its amino acid sequence, pharmaceutical preparation, and effective dose. It blocks inflammatory processes and neutralizes the biological activity of TNF‐α, thereby achieving the desired therapeutic effect. Comparative studies of quality, structure, stability, pharmacodynamics, pharmacokinetics, and safety in preclinical studies have shown that HS016 is consistent across batches for all key quality indicators and highly similar to adalimumab (H. Wang, Hisun BioRay Bio‐Pharmaceutical Co., Ltd, unpublished work). There is no biosimilar equivalent of adalimumab marketed in China at present, but HS016 might be an alternative with a high benefit/risk ratio to facilitate treatment in a broader range of patients.

Thus, based on preclinical studies, a phase 1 study in Chinese healthy male subjects who received a single subcutaneous injection of HS016 or the originator adalimumab was conducted to compare the equivalence of HS016 to the reference originator adalimumab, with emphasis on pharmacokinetic properties, safety, and immunogenicity.

## Methods

### Study Subjects

Chinese healthy male subjects (age range 18‐40 years) were required to weigh between 50 and 70 kg (inclusive), with a body mass index (BMI) of 20–25 kg/m^2^ (inclusive) on initial screening. During the study period, subjects (or their spouses) used appropriate and effective contraceptive measures such as abstinence, oral contraceptives, intrauterine devices and condoms combined with a contraceptive diaphragm. Subjects were excluded if they had systemic diseases such as mental, respiratory, cardiovascular, digestive, urinary, reproductive, skeletal motor, hematological, endocrine, or neurological conditions. Additional details of patient eligibility criteria are provided in supplementary Appendix 1.

### Study Design

This was a randomized, parallel‐group, double‐blinded phase 1 clinical trial conducted at Huashan Hospital of Fudan University. Our study in humans closely followed the Declaration of Helsinki principles and complied with the Good Clinical Practices and Provisions for Drug Registration issued by the National Medical Products Administration. The ethics committee of Huashan Hospital of Fudan University approved the study protocols (No. 2016‐213). All subjects who participated in the study signed informed consent documents before being enrolled. This trial was registered with the Chinese Clinical Trial Registry (Number ChiCTR1900025858).

The aim was to enroll a total of 136 Chinese healthy male subjects in the trial, and eventually 122 successfully met the enrollment criteria; 61 were assigned to the HS016 group and 61 to the originator adalimumab group. If more than 14 subjects dropped out before completion of all clinical measurements, additional subjects were enrolled until 122 subjects completed the entire trial. Subjects were randomly assigned at a 1:1 ratio to receive a subcutaneous injection of 40 mg/0.8 mL of either HS016 or adalimumab on day 1. Subjects were discharged from the clinical pharmacology unit (CPU) on day 3 and were required to return to the CPU over the next days for safety evaluations and blood sampling for pharmacokinetic and human‐antihuman antibody (HAHA) assessments, until the end of the study period (day 70). Plasma samples for pharmacokinetic assessments were collected at predose (within 2 hours), and at 6 and 12 hours, and 1, 2, 3, 4, 5, 6, 7, 8, 9, 14, 21, 28, 35, 42, 56, and 70 days postdose. HAHA tests were performed at prespecified visits including day 1 (predose) and days 14, 42, and 70. Neutralizing antibody (Nab) was detected in subjects with positive HAHAs.

### Pharmacokinetic Evaluations

Pharmacokinetic evaluations included the area under the concentration‐time curve (AUC) from time 0 to the last detectable drug concentration (AUC_0‐t_), the AUC from time 0 extrapolated to infinity (AUC_0‐∞_), the maximum plasma concentration (C_max_), time to C_max_, apparent clearance (CL/F), elimination half‐life (T_½_), and plasma concentrations.

### Safety Evaluations

Safety evaluations included measurements of vital signs, clinical laboratory data, other medications used, the incidence and type of treatment‐emergent adverse event (TEAEs), serious AEs (SAEs) or TEAEs related to the study drug, which prevented the subject from completing the trial. AE reporting was used to assess safety and all reported terms for AEs were coded according to the *Medical Dictionary for Regulatory Activities* (*MedDRA* v 19.1).

### Immunogenicity Evaluations

Immunogenicity evaluations included the number and percentage of subjects who were HAHA‐positive (negative) or Nabs‐positive (negative) at each visit after drug administration to each group.

### Bioanalytical Methods

Plasma concentrations of HS016 and adalimumab were determined using an enzyme‐linked immunosorbent assay (ELISA) that was methodologically validated. The ELISA plate was precoated with recombinant TNF‐α, sealed, and incubated with quality‐control samples and the experimental drug. After excess samples had been washed away, horseradish peroxidase (HRP)‐labeled human antiadalimumab was added to form the “antigen‐drug‐antibody” complex, and a color reaction was elicited by adding the HRP‐labeled substrate 3,3′,5,5′‐Tetramethylbenzidine (TMB), which produces a response proportional to the HS016 concentration. The optical density values were detected by the dual‐wavelength method, using a detection wavelength of 450 nm and a reference wavelength of 630 nm. The standard curve was fitted by a 4‐Parameter model, with a weight of 1/Y^2^. The lower limit of quantification (LLOQ) was 15.625 ng/mL, and all plasma concentrations of subjects <LLOQ were recorded as below quantification limit (BQL) in the calculation of pharmacokinetic parameters.

HAHA status was determined using the bridging electrochemiluminescence (ECL) immunoassay based on the meso scale discovery ECL platform, which consisted of screening and immunosuppression confirmatory assays. Nab status was determined based on the principle that L‐929 cells were highly sensitive to the killing and inhibition of recombinant human TNF activity under the action of actinomycin D.^6^

### Statistical Analyses

The cohort size was determined according to earlier studies on the bioavailability of adalimumab. For an 80% power to ensure that all end points met the equivalence at the same time, according to Bonferroni, there should be a 90% power for each end point. We assumed that the coefficient of variation (CV) of AUC_0‐t_ would be the same as the AUC_0‐∞_, and the true ratio of AUC_0‐t_ between the experimental (HS016) and control (adalimumab) groups was 1 ± 0.05, based on a CV% of 27.7% (AUC_0‐360_ for adalimumab) and 90% power. Thus, a total of 88 subjects (44 per group) was needed for the trial. We assumed that true ratio of C_max_ between HS016 and the control (adalimumab) groups was 1 ± 0.05, and based on the CV% of 33.0% (C_max_ for adalimumab) and 90% power, 122 subjects (61 subjects per group) were to be enrolled. Finally, the larger cohort size among end points was selected (61 per group), allowing for a dropout rate of 10% for pharmacokinetic measurements; 136 subjects were needed for randomization.

Pharmacokinetic parameters were calculated using a noncompartment model (WinNonLin ver 6.4, Certara Corp, Princeton, New Jersey), and all BQL were represented as 0 in the pharmacokinetic parameters and plasma concentration‐time profiles. Pharmacokinetic equivalence between the 2 groups was determined by comparison of the 90%CIs for the geometric mean (GM) test‐to‐reference ratios of the AUC_0‐t_, AUC_0‐∞_, and C_max_, with the bioequivalence criteria range (80% to 125%) specified above.

According to the principle of intention to treat, the full analysis set was defined as the group of randomized subjects who were given the experiment drug and had at least 1 available postadministration plasma concentration measurement, mainly for the statistical description of the trial population. The pharmacokinetic population included all subjects who had at least 1 pharmacokinetic profile that could be evaluated in the full analysis set and who completed the trial without a significant program deviation that could have affected the pharmacokinetic evaluation. The statistical descriptions of pharmacokinetic evaluations were all based on the pharmacokinetic population. If necessary, a small number of subjects (the pharmacokinetic parameters may be affected by the deviation of protocol) were removed for sensitivity analysis. Plasma concentrations and pharmacokinetic parameters were summarized between the 2 groups using quantitative descriptive statistics (and by time points for plasma concentrations), and a LLOQ was highlighted in the plasma concentration‐time curve.

All randomized subjects who received the experimental drugs were included in the safety set used for safety and immunogenicity analysis.

All statistical analysis was carried out using SAS (ver 9.2, SAS Institute, Cary, North Carolina). Statistical t‐tests and chi‐squared tests were used to assess categorical and continuous variables. The natural logarithmic transformation of AUC_0‐t_, AUC_0‐∞_, and C_max_ was performed using an ANOVA model with a fixed effect and least‐squares GM and a 95%CI ratio for each group; the ratio of the least‐squares GM and 90%CI between the 2 groups was calculated. All tests were 2‐sided, and *P* < .05 was deemed to be a significant difference.

## Results

### Subjects’ Disposition and Features

A total of 795 subjects were screened, with a screening rate of failure 661 subjects (of these, 2 did not meet the inclusion criteria, but they actually received the drug and completed the trial). Finally, 136 subjects were enrolled and completed the trial (68 subjects per group). All randomized subjects were included in the full‐analysis, pharmacokinetic, and safety set populations. The disposition of subjects is shown in Figure S1. Most subjects’ demographic and baseline characteristics were similar between the 2 groups except for height (Table 1). The subjects were young, with an age range of 18‐37 years and BMIs of 20‐25 kg/m^2^.

**Table 1:** Summary of Demographic Data and Baseline Characteristics Between Biosimilar HS016 and Originator Adalimumab

Characteristics	HS016 (n = 68)	Adalimumab (n = 68)	*P*‐value	Total (n = 136)
Mean age, y (range)	26.3 (18‐36)	25.5 (18‐37)	0.205	25.9 (18‐37)
Nationality, n (%)			> 0.999	
Han	65 (95.6%)	64 (94.1%)		129 (94.9%)
Others	3 (4.4%)	4 (5.9%)		7 (5.1%)
Mean height, cm (range)	168.0 (157‐182)	170.1 (157‐184)	0.033	169.0 (157‐184)
Mean weight, kg (range)	62.6 (53.0‐70.0)	63.0 (50.6‐70.0)	0.564	62.8 (50.6‐70.0)
Mean BMI, kg/m^2^ (range)	22.2 (20.0‐25.0)	21.8 (20.0‐24.8)	0.069	22.0 (20.0‐25.0)
Mean CRP level,^a^ mg/L (range)	3.3 (3.0‐4.1)	3.3 (3.1‐3.4)	0.958	3.3 (3.0‐4.1)
Mean ESR level, mm/h (range)	3.5 (2.0‐13.0)	3.7 (2.0‐11.0)	0.549	3.6 (2.0‐13.0)

BMI indicates body mass index; CRP, C‐reactive protein; ESR, erythrocyte sedimentation rate.

a Mean CRP levels at screening (days –14 to –2).

### Pharmacokinetics

**Figure 1:**
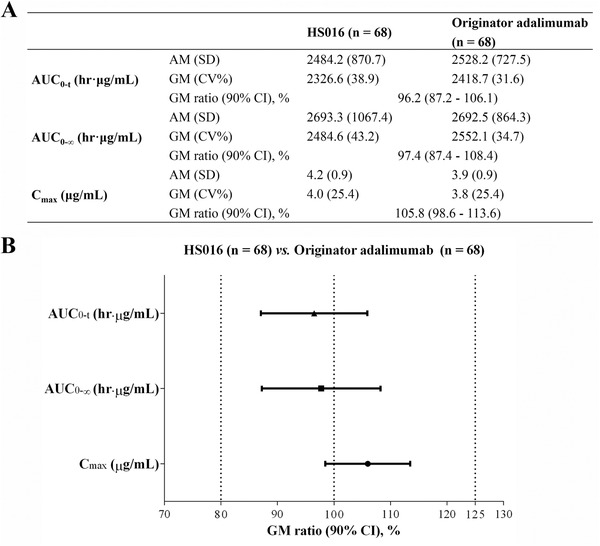
GM (CV%) (A) and Forest plot (B) showing point estimates and the 90%CIs of AUC_0‐t_, AUC_0‐∞_, and C_max_ for HS016 and adalimumab (bioequivalence was declared if the 90%CIs were within the prespecified margins of 80% to 125%). AM indicates arithmetic mean; AUC_0‐t_, the area under the plasma concentration‐time curve (AUC) from time 0 to the last detectable drug concentration; AUC_0‐∞_, AUC from time 0 extrapolated to infinity; C_max_, maximum plasma concentration; CV, coefficient of variation; GM, geometric mean.

The GMs of AUC_0‐t_, AUC_0‐∞_, and C_max_ in male healthy subjects were similar between the HS016 (2326.6 h·μg/mL, 2484.6 h·μg/mL, and 4.0 μg/mL, respectively) and adalimumab (2418.7 h·μg/mL, 2552.1 h·μg/mL, and 3.8 μg/mL) groups (Figure 1A). The 90%CIs of AUC_0‐t_ (87.2% to 106.1%), AUC_0‐∞_ (87.4% to 108.4%), and C_max_ (98.6–113.6%) were all within the prespecified bioequivalence criteria of 80% to 125% (Figure 1B). This finding confirmed the pharmacokinetic equivalence between HS016 and adalimumab.

**Figure 2:**
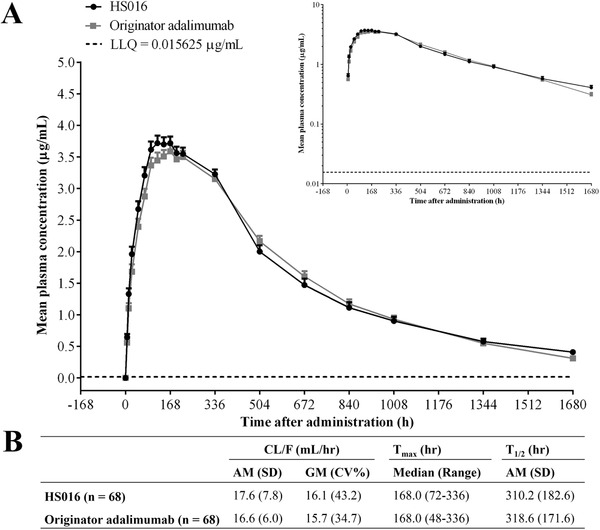
A, Mean plasma concentration‐time profiles on linear and semilogarithmic scales. B, T_max_, CL/F, and T_½_ in Chinese healthy male subjects following a single subcutaneous injection of 40 mg of either HS016 or adalimumab. AM indicates arithmetic mean; CL/F, total clearance; CV, coefficient of variation; GM, geometric mean; LLQ, lower limit of quantitation; T_max_, time of maximum plasma concentration after administration; T_½_, elimination half‐life.

From Figure 2A, it is clear that the mean plasma concentration‐time profiles of male healthy subjects after a single subcutaneous injection of HS016 or adalimumab basically overlapped over time. The mean plasma concentration reached a peak 168 hours after administration of HS016 and adalimumab (3.7 versus 3.6 μg/mL, respectively) and then declined monophasically. After a single subcutaneous injection of 40 mg of either HS016 or adalimumab, the overall values of the other pharmacokinetic parameters (T_max_, CL/F, T_½_) were similar between the 2 groups (Figure 2B).

### Safety

The incidence of TEAEs was similar in the HS016 and adalimumab groups (92.6% versus 95.6%). There were no SAEs related to the experimental drugs and no TEAEs leading to discontinuation/withdrawal of treatment or death. Only 1 subject (1.5%) in the HS016 group experienced a SAE, which was unrelated to the experimental drug (Table 2).

**Table 2:** Summary of TEAEs (Safety Population) Between HS016 and Originator Adalimumab Groups

	HS016 (n = 68)		Adalimumab (n = 68)	
	Events	n (%)	Events	n (%)
TEAEs	288	63 (92.6)	265	65 (95.6)
Mild	283	60 (88.2)	265	65 (95.6)
Moderate	5	3 (4.4)	0	0
Severe	0	0	0	0
Drug‐related TEAEs	172	50 (73.5)	148	55 (80.9)
SAEs	1	1 (1.5)	0	0 (0.0)
TEAE leading to discontinuation	0	0 (0.0)	0	0 (0.0)
Deaths	0	0 (0.0)	0	0 (0.0)
Drug‐related TEAE reported in >5% of subjects in any treatment group				
Examinations	124	43 (63.2)	108	46 (67.6)
Monocyte percentage increased	16	14 (20.6)	10	9 (13.2)
Fibrinogen decreased	14	14 (20.6)	17	16 (23.5)
Neutrophil count decreased	10	10 (14.7)	8	7 (10.3)
Neutrophil percentage decreased	10	8 (11.8)	19	14 (20.6)
Neutrophil count increased	8	8 (11.8)	3	3 (4.4)
Leukocyte count increased	7	7 (10.3)	3	3 (4.4)
Positive antinuclear antibodies	7	7 (10.3)	10	10 (14.7)
Lymphocyte percentage decreased	8	7 (10.3)	1	1 (1.5)
Alanine aminotransferase increased	6	5 (7.4)	3	3 (4.4)
Lymphocyte percentage increased	6	5 (7.4)	10	7 (10.3)
CRP increased	5	4 (5.9)	1	1 (1.5)
Hemobilirubin increased	4	4 (5.9)	5	5 (7.4)
Systemic diseases and administration site conditions	13	12 (17.6)	11	7 (10.3)
Fever	6	6 (8.8)	2	2 (2.9)
Erythema at injection site	6	6 (8.8)	5	5 (7.4)
Respiratory, thoracic, and mediastinal diseases	12	8 (11.8)	7	5 (7.4)
Nasal congestion	4	4 (5.9)	1	1 (1.5)
Skin and subcutaneous tissues disease	6	5 (7.4)	8	7 (10.3)
Gastrointestinal diseases	6	4 (5.9)	7	5 (7.4)
Infections and infestations	3	3 (4.4)	5	4 (5.9)

CRP indicates C‐reactive protein; SAE, serious adverse event; TEAE, treatment‐evoked adverse event.

A total of 172 cases of drug‐related TEAEs occurred in 50 subjects (73.5%) in the HS016 group and 108 in 46 subjects (67.6%) in the adalimumab group. The most frequently reported drug‐related TEAEs were monocyte percentage count increases, a fibrinogen decrease, neutrophil count decreases or increases, neutrophil percentage decreases, leukocyte count increases, positive antinuclear antibodies, lymphocyte percentage decreases or increases, alanine aminotransferase increases, C‐reactive protein and hemobilirubin increases, systemic diseases, and reactions at the site of drug administration.

Among the 288 TEAE cases, 63 (92.6%) occurred in subjects in the HS016 group. Of these, 283 TEAE cases occurred in 60 (88.2%) subjects and were mild, whereas 5 cases occurred in the remaining 3 (4.4%) subjects that were moderate. No severe TEAEs occurred.

In the adalimumab group 265 TEAEs were reported in 65 (95.6%) subjects and all were mild in nature. The TEAEs with positive antinuclear antibodies in the HS016 and adalimumab groups were also mild (7 [10.3%] versus 10 [14.7%]).

Laboratory tests, vital signs, physical examination, electrocardiogram, serum virology, rheumatic immunity, and chest x‐ray examination results were evenly distributed between the 2 groups, with no significant changes after medication.

### Immunogenicity

**Figure 3:**
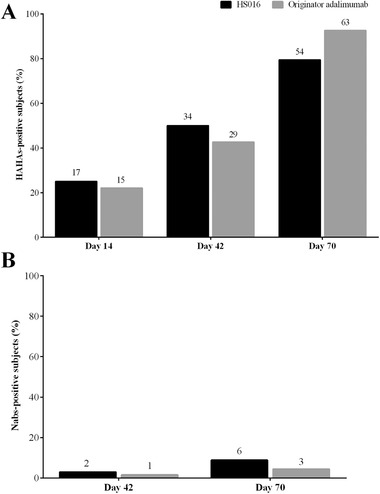
Development of (A) HAHAs and (B) Nabs in Chinese healthy male subjects after a single 40‐mg subcutaneous injection of HS016 or adalimumab on days 14, 42, and 70 between the 2 groups (safety population). HAHA indicates human antihuman antibody; NAb, neutralizing antibody.

There were no HAHA‐positive participants at baseline, and all HAHA‐positive cases occurred after a single subcutaneous injection of HS016 or adalimumab. Overall, 17 (25.0%) and 15 (22.1%) subjects developed antibodies to adalimumab in the HS016 and adalimumab groups at day 14 and day 34 (50.0%), and 29 (42.6%) subjects developed antibodies to adalimumab after 42 days. HAHA positivity was detected in 54 (79.4%) and 63 (92.6%) subjects, respectively, at any time point during the study (including at 70 days). The proportion of HAHA‐positive cases appeared to increase with time, but there was no significant difference between the 2 groups. Confirmed HAHA‐positive samples were further analyzed for their neutralizing capacity, and we found that all subjects in the HS016 and adalimumab groups were Nabs‐negative after 14 days. After 42 days, 2.9% (n = 2) and 1.5% (n = 1) of the subjects were Nab‐positive in the HS016 and adalimumab groups, respectively. By the end of the study, the percentage of Nab‐positive subjects in the treatment groups had risen to 8.8% (n = 6) and 4.4% (n = 3) for HS016 and adalimumab, respectively (Figure 3).

In a subgroup analysis the impact of HAHAs and Nabs on the pharmacokinetic parameters was evaluated (Table S1). All pharmacokinetic parameters were similar between the 2 groups, regardless of different HAHA and Nab statuses (positive or negative). The GMs of AUC_0‐t_, AUC_0‐∞_, C_max_, and CL/F in HAHA‐positive subjects appeared to be lower than those in HAHA‐negative subjects. Similar results were found for the Nab subgroups. The pharmacokinetic effect of HAHA and Nabs positivity was mainly reflected in the mean T_½_, which was shortened in both the HS016 (245.6 versus 554.8 hours in HAHAs and 97.6 versus 261.0 hours in Nabs) and the adalimumab (296.7 versus 593.7 hours in HAHAs and 115.0 versus 303.5 hours in Nabs) groups.

## Discussion

The present phase 1 trial was designed to evaluate equivalent efficacy regarding the pharmacokinetic parameters (AUC_0‐t_, AUC_0‐∞_, and C_max_) and also to compare safety and immunogenicity in male healthy subjects after a single injection of 40 mg of either HS016 or adalimumab. In order to ensure the homogeneity of the study population and better reflect the pharmacokinetic differences between the HS016 and adalimumab groups, we selected healthy male subjects for the study and limited the inclusion criteria, such as to a negative HAHA status and BMIs between 20 and 25 kg/m^2^.^11^ We used a single subcutaneous injection of 40 mg HS016 because it is the recommended therapeutic dose of adalimumab for patients with AS or RA and was an acceptable dosage for healthy subjects.^6^, ^12^

The GMs of the pharmacokinetic parameters were all similar between the 2 groups, and the 90%CI ratios of AUC_0‐t_, AUC_0‐∞_, and C_max_ were fully contained within the standard prespecified criteria of 80% to 125%, which indicated subcutaneous comparability between HS016 and adalimumab. A total of 5 subjects (2 HS0116, 3 adalimumab) were excluded from pharmacokinetic sensitivity analysis. The pharmacokinetic parameters were similar, and the 90%CI of the GM ratios were all within the bioequivalence margin between the pharmacokinetic population and the pharmacokinetic‐sensitive population, indicating that elimination of outliers had little impact on pharmacokinetic analysis (data not shown).

The overall safety was similar between the HS016 and adalimumab groups, indicating that Chinese healthy male subjects exhibited good tolerance after a single subcutaneous injection of 40 mg of HS016 or adalimumab. The severity of side effects and TEAEs in the HS016 and adalimumab groups were generally similar and mostly mild, and only 3 subjects in the HS016 group experienced moderate adverse events. With the wide application of TNF‐α inhibitors in the clinic, more and more attention has been paid to their potential associated risks. SAEs have been reported including malignant tumors and bacterial, viral, and fungal infections,^13^, ^14^ all related to blockade of the physiological effects of TNF‐α. Only a few cases of infections (4.4% versus 5.9%) and no malignant tumor cases were found during our study. A meta‐analysis also confirmed that even though many infectious AEs were detected in the TNF‐α group, there was still no significant difference in serious infection events among the patients with/without TNF‐α therapy; also there was no increased risk of malignancy associated with TNF‐α inhibitor therapy.^15^ In this trial the most frequent TEAEs that occurred in the HS016 and adalimumab groups were all types of indicator examinations (86.8% versus 89.7%), which were different from other biosimilar studies.^10^, ^16^, ^17^, ^18^ Thus, the higher incidence of the indicator examinations may have been the reason for the higher incidence of TEAEs (HS016, 92.6% versus adalimumab, 95.6%).

The immunogenicity of HS016 was also similar to that of adalimumab. Although the HAHA‐positive subject proportion in the HS016 group (79.4%) was lower than that in the adalimumab group (92.6%) (*P* = .026), there was no significant difference in the absolute number of subjects (9 cases). A larger cohort size will be required for further validation of these findings. By the end of the study, the percentages of Nab‐positive subjects in the HS016 and adalimumab groups were 8.8% (n = 6) and 4.4% (n = 3) (*P* = .3), respectively, which was similar to those reported in earlier studies.^6^, ^12^

The production of HAHAs affects efficacy through pharmacokinetic actions, which was also associated with an increased frequency of both major and minor clinical AEs, such as infusion reactions.^19^, ^20^, ^21^ Consistent with an earlier study, the influence of HAHAs‐positivity on pharmacokinetic parameters was mainly reflected in T_½_, which was shortened in both the HS016 and adalimumab groups.^10^ Similar results were found for Nabs positivity, although Nabs‐positive subjects were rare in both the HS016 and adalimumab groups, with only 6 and 3 cases detected, respectively.

## Conclusions

In this phase 1 trial, after a single subcutaneous injection of HS016 (a biosimilar of adalimumab) or adalimumab in Chinese healthy male subjects, the pharmacokinetic evaluations (AUC_0‐t_, AUC_0‐∞_, and C_max_) were equivalent for the 2 groups, with similar pharmacokinetic characteristics and safety and immunogenicity data. HS016 can be used to confirm comparability with adalimumab in future studies involving AS patients.

## Conflicts of Interest

The authors declare no conflicts of interest.

## Data‐Sharing Statement

The data sets used and/or analyzed during the current study are available from the corresponding author on reasonable request.

## Supporting information



Supplemental Information
