# Supplementary material for: A Randomized, Double‐Blind, Parallel‐Group, Phase 1 Clinical Trial Comparing the Pharmacokinetic, Safety, and Immunogenicity of the Biosimilar HS016 and the Originator Adalimumab in Chinese Healthy Male Subjects
Source: Clin Pharmacol Drug Dev. 2020 May 28;10(3):317–25. doi: 10.1002/cpdd.816 (PMC7984335; doi:10.1002/cpdd.816)
Supplement: Supplementary file 1 — Supplemental Information [file CPDD-10-317-s001.docx]

**Supplementary materials**

**Table S1. Summary of pharmacokinetic parameters for biosimilar HS016 and originator adalimumab in HAHAs or Nabs subgroups**

|  | **AUC_0-t_ (hr·μg/mL)** | | **AUC_0-∞_ (hr·μg/mL)** | | **C_max_ (μg/mL)** | | **CL/F (mL/h)** | | **T_max_ (hr)** | **T_1/2_ (hr)** |
| --- | --- | --- | --- | --- | --- | --- | --- | --- | --- | --- |
|  | **AM (SD)** | **GM (CV%)** | **AM (SD)** | **GM (CV%)** | **AM (SD)** | **GM (CV%)** | **AM (SD)** | **GM (CV%)** | **Median (range)** | **AM (SD)** |
| **HAHAs-positive** |  |  |  |  |  |  |  |  |  |  |
| HS016 (n = 54) | 2236.7 (731.6) | 2113.5 (36.0) | 2352.5 (824.0) | 2207.9 (38.1) | 4.0 (0.9) | 3.9 (25.5) | 19.4 (7.7) | 18.1 (38.1) | 168.0 (72-336) | 245.6 (143.2) |
| Originator adalimumab (n = 63) | 2491.0 (729.9) | 2380.9 (31.8) | 2628.4 (854.1) | 2491.2 (34.6) | 3.9 (0.9) | 3.8 (25.3) | 17.0 (6.0) | 16.1 (34.6) | 168.0 (48-336) | 296.7 (156.7) |
| **HAHAs-negative** |  |  |  |  |  |  |  |  |  |  |
| HS016 (n = 14) | 3439.0 (698.9) | 3370.0 (21.5) | 3983.2 (896.4) | 3884.8 (24.0) | 4.6 (1.1) | 4.5 (22.3) | 10.6 (2.6) | 10.3 (24.0) | 120.0 (72-216) | 554.8 (76.3) |
| Originator adalimumab (n = 5) | 2997.3 (556.2) | 2949.4 (21.1) | 3499.7 (570.5) | 3458.3 (17.9) | 3.9 (1.0) | 3.7 (29.6) | 11.7 (2.3) | 11.6 (17.9) | 192.0 (96-216) | 593.7 (102.1) |
| **Nabs-positive** |  |  |  |  |  |  |  |  |  |  |
| HS016 (n = 6) | 1652.9 (237.9) | 1638.0 (15.0) | 1739.0 (183.8) | 1730.9 (10.9) | 4.3 (0.8) | 4.3 (18.5) | 23.2 (2.6) | 23.1 (10.9) | 192.0 (96-336) | 97.6 (26.3) |
| Originator adalimumab (n = 3) | 1399.3 (331.4) | 1374.9 (22.9) | 1417.6 (339.3) | 1392.4 (23.1) | 3.9 (1.0) | 3.8 (25.5) | 29.2 (6.2) | 28.7 (23.1) | 168.0 (120-216) | 115.0 (28.0) |
| **Nabs-negative** |  |  |  |  |  |  |  |  |  |  |
| HS016 (n = 48) | 2309.6 (740.7) | 2181.9 (36.6) | 2416.5 (839.1) | 2264.6 (39.1) | 4.0 (0.9) | 3.9 (26.2) | 19.0 (7.9) | 17.7 (39.1) | 168.0 (72-336) | 261.0 (141.6) |
| Originator adalimumab (n = 59) | 2536.2 (704.0) | 2437.4 (29.6) | 2676.3 (828.5) | 2552.1 (32.3) | 3.9 (0.9) | 3.8 (25.7) | 16.5 (5.4) | 15.7 (32.3) | 168.0 (48-336) | 303.5 (155.3) |

Note. AM, arithmetic mean; AUC_0-t_, the area under the plasma concentration-time curve (AUC) from time zero to the last detectable drug concentration; AUC_0-∞_, AUC from time zero extrapolated to infinity; C_max_, maximum plasma concentration; CL/F, total clearance; CV, coefficient of variation; GM, geometric mean; HAHA, human anti-human antibody; NAb, neutralizing antibody; SD, standard deviation; T_max_, time of maximum plasma concentration after administration; T_1/2_, elimination half-life time.

**
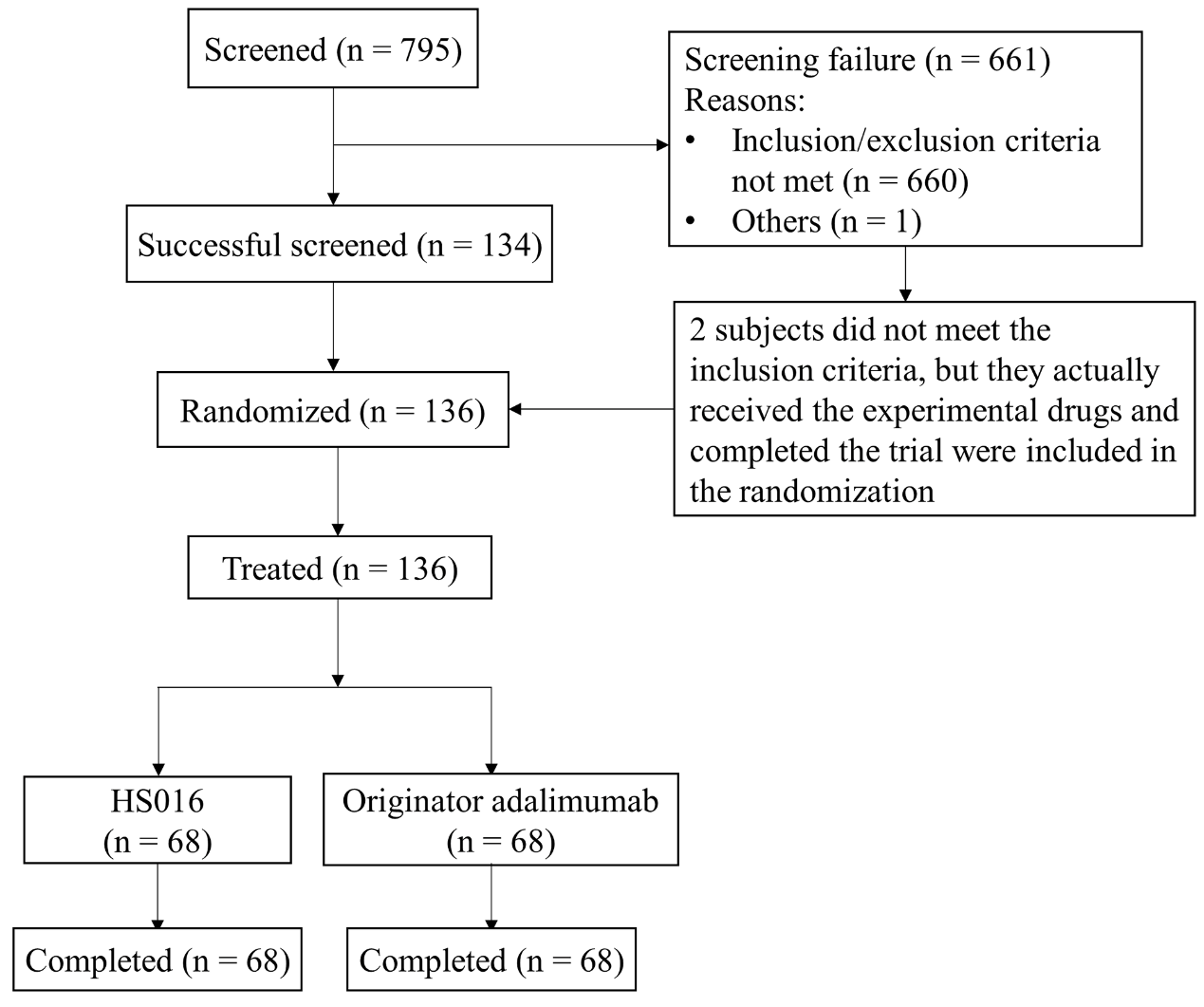
**

**Figure S1. Scheme of subjects screened and their enrolling disposition in the study**

**Appendix 1. Eligibility criteria**

**Inclusion criteria**

Subjects must have met all of the following inclusion criteria to be enrolled in the study:

1. Must have signed and dated written informed consent before inclusion, and must have been able to comply with the requirements of the entire study

2. Males, aged 18 to 40 years old (inclusive, the date of signing the informed consent) shall prevail;

3. Body mass index (BMI) between 20–25 kg/m^2^ (inclusive values) and body weight between 50 kg and 70 kg (inclusive values).

4. The subject or spouse promised to use effective methods of contraception during the entire trial period, such as abstinence, oral contraceptives, intrauterine devices or dual barrier methods (such as condoms plus contraceptive diaphragms).

**Exclusion criteria**

Subjects meeting any of the following criteria were not enrolled in the study:

1. Subjects with mental, respiratory, cardiovascular, digestive, urinary, reproductive, skeletal and motor, hematological, endocrine or nervous system diseases;

2. Subjects who had severe bleeding that could affect venous blood collection;

3. Subjects with skin diseases or others that may affect subcutaneous injection;

4. Subjects with a history of malignant tumors;

5. Subjects known to have had an active bacterial, viral, fungal, parasitic or other infection or any infection requiring antibiotic treatment or hospitalization within the first 4 weeks of enrollment; or those with the following symptoms: fever, night sweats, chills, weight loss, feeling tired, muscle pain, cough, shortness of breath, bloody sputum, diarrhea, stomach ache, a burning sensation during urination or more frequent urination than normal; subjects with red, hot skin or bedsore in the body; subjects with severe skin infections, including multiple folliculitis.

6. Subjects who had undergone surgery within two months prior to signing the informed consent form;

7. Alcoholics or those who regularly drank alcohol within 3 months of the trial starting, that is, drinking more than 14 units of alcohol per week (14 bottles of 360 mL beer or 630 mL of 40% alcohol or 2,100 mL of wine), with positive alcohol breath test measurements;

8. Subjects who had used soft drugs (such as marijuana) within 3 months prior to the signing of informed consent forms or who had taken hard drugs (such as cocaine, phencyclidine, etc.) within 1 year of the trial starting; subjects with positive drug abuse test results (cocaine, marijuana, morphine, amphetamine, methamphetamine, phencyclidine, benzodiazepines, barbiturates, methadone and tricyclic antidepressants);

9. A heavy smoker or a subject who had smoked > 5 cigarettes per day for 3 months prior to the signing of informed consent;

10. Subjects with a positive nicotine result 1 day (-1 d) before enrollment;

11. Had a history of drug or food allergies, or had special dietary requirements, could not comply with a unified diet or a history of specific allergic reaction (asthma, rubella, eczematous dermatitis); subjects known to be allergic to any of the components of the experimental drug formulation or emulsion (contained in the needle cap of the injection syringe);

12. Subjects who consumed excessive amounts of tea, coffee and/or caffeinated beverages (more than 8 cups, 1 cup = 250 mL) daily;

13. Subjects who received any medical treatment (including prescription drugs, non-prescription drugs, biological products, proprietary Chinese medicine, vitamins, dietary supplements, etc.) and healthcare products within 14 days of signing informed consent;

14. Subjects who used traditional Chinese medicine (except proprietary Chinese medicine) and health care products during the trial;

15. Subjects who had a blood donation history within 3 months prior to the signing of the informed consent form, or planned to donate blood during the trial;

16. Subjects who may receive any biological drugs 3 months after administration of the experimental drug or planned to receive monoclonal antibody drugs within 9 months;

17. Subjects who planned to donate sperm within 6 months after administration of the experimental drug;

18. Subjects who planned to undergo surgery (including plastic surgery, dental surgery, and oral surgery) during the trial;

19. Subjects who planned to participate in strenuous exercise during the trial, including physical contact or collisional sports;

20. Subjects with positive results for the human anti-human antibodies (HAHAs) test;

21. Subjects who had participated in other clinical trials within 3 months prior to signing informed consent;

22. Subjects who received any vaccine within 6 months prior to the signing of informed consent form;

23. Subjects who failed to meet the health standards in a comprehensive physical examination including: abnormal vital signs (pulse rate in the waking state < 50 beats/min or > 100 beats/min, systolic blood pressure ≥ 140 mmHg or diastolic blood pressure ≥ 90 mmHg, axillary temperature > 37.2°C), had a clinically significant abnormal laboratory results for routine blood, urine, liver and renal functions (in which albumin, blood lymphocytes, monocytes, CRP and erythrocyte sedimentation rates must be within normal values); had a clinically significant abnormal electrocardiogram examination; PR interval ≥ 210 ms, QRS ≥ 120 ms, QTc ≥ 450 ms, or had a previous history of abnormal electrocardiograms;

24. Subjects with low-density lipoprotein cholesterol > 3.36 mmol/L during the screening period, or with a higher fasting blood glucose level than the normal range during the screening period and admission;

25. Subjects with positive results for human immunodeficiency virus (HIV) antibody, hepatitis B surface antigen, hepatitis B core antibody, anti-hepatitis C virus or in the treponema pallidum antibody test;

26. Subjects with positive results for rheumatoid factor (RF), anti-double strand DNA antibody (anti-dsDNA), antinuclear antibody (ANA) or anti-cyclic citrullinated peptide antibody (anti-CCP) tests;

27. Subjects who had previously been infected or latently infected with mycobacterium tuberculosis (with positive results in the interferon-gamma release assay (T-SPOT));

28. Subjects that the investigator considered unsuitable for enrollment or may not have been able to complete the trial for various reasons;

29. Researchers, employees or related personnel of research centers, sponsors, and contract research organizations.
